# Ethnic Pharmacogenomic Differences in the Management of Asian Patients with Metastatic Prostate Cancer

**DOI:** 10.3390/cancers14020407

**Published:** 2022-01-14

**Authors:** Darren M. C. Poon, Kuen Chan, Tim Chan, Foo-Yiu Cheung, Daisy Lam, Martin Lam, Ka-Suet Law, Conrad Lee, Eric K. C. Lee, Angus Leung, Henry Sze, Chi-Chung Tong, Kenneth C. W. Wong, Philip Kwong

**Affiliations:** 1 Department of Clinical Oncology, State Key Laboratory of Translational Oncology, Sir YK Pao Centre for Cancer, Hong Kong Cancer Institute, The Chinese University of Hong Kong, Hong Kong; lcm306@ha.org.hk (D.L.); kennethcw.wong@cuhk.edu.hk (K.C.W.W.); 2 Comprehensive Oncology Centre, Hong Kong Sanatorium & Hospital, Hong Kong; 3 Pamela Youde Nethersole Eastern Hospital, Hong Kong; chank1@ha.org.hk; 4 Queen Elizabeth Hospital, Hong Kong; ctw263@ha.org.hk; 5 Hong Kong Integrated Oncology Centre, Hong Kong; Fycheung@hkioc.com.hk (F.-Y.C.); pkwong@hkioc.com.hk (P.K.); 6 United Christian Hospital, Hong Kong; lhc425@ha.org.hk; 7 Princess Margaret Hospital, Hong Kong; lks174a@ha.org.hk; 8 Hong Kong International Medical Centre, Hong Kong; conradcylee@hotmail.com; 9 Tuen Mun Hospital, Hong Kong; leekc4@ha.org.hk; 10 AMO Oncology Centre, Hong Kong; anguskcleung@amo-oncology.com.hk; 11 HEAL Oncology Centre, Hong Kong; henry@heal-oncology.com; 12 Queen Mary Hospital, Hong Kong; tccz01@ha.org.hk

**Keywords:** Asians, chemotherapy, metastasis, pharmacogenomics, prostate cancer, taxane

## Abstract

**Simple Summary:**

With the recognition that ethnicity may influence prognosis and outcomes, there are ongoing controversies over how best to treat patients with prostate cancer. This review discusses recent evidence for the impacts of Asian ethnicity on metastatic prostate cancer treatment.

**Abstract:**

Progression to metastatic disease occurs in about half of all men who develop prostate cancer (PC), one of the most common cancers in men worldwide. Androgen deprivation therapy has been the mainstay therapy for patients with metastatic PC (mPC) since the 1940s. In the last decade, there has been unprecedented advancement in systemic therapies, e.g., taxane, androgen-signalling pathway inhibitors, and biomarker-driven targeted therapies for various stages of disease, resulting in overall survival improvement. Adding to ongoing controversies over how best to treat these patients is the recognition that ethnicity may influence prognosis and outcomes. This review discusses recent evidence for the impacts of Asian ethnicity specifically, which includes environmental, sociocultural, and genetic factors, on the approach to pharmacological management of mPC. Clear inter-ethnic differences in drug tolerability, serious adverse events (AEs), and genetic heterogeneity must all be considered when dosing and scheduling for treatment, as well as designing future precision studies in PC.

## 1. Introduction

Prostate cancer (PC) is the second most common cancer in men, and the fifth leading cause of death worldwide, with steadily declining mortality rates that are likely due to advances in early detection and treatment [1]. While treatment options for metastatic PC (mPC) are expanding, an understanding of the ethnic diversity of this global condition may help to further refine treatment planning and clinical management.

For example, a retrospective analysis of U.S. Surveillance, Epidemiology and End Results (SEER) registry data observed a difference in overall survival (OS) between Asian men diagnosed with distant, *de novo* metastatic hormone-sensitive PC (mHSPC), when compared with those of other ethnicities (30 vs. 24−25 months, *p* < 0.001) [2]. The authors noted that similar outcomes were observed for Asian populations in America and Asia (in other studies), suggesting that biological factors might have played a role in responsiveness to therapy. A retrospective analysis of 20-year data from East London’s Bartholomew’s Hospital observed longer survival in Black versus White men with metastatic castration-resistant PC (mCRPC), who received hormone-based therapy only (39.7 months vs. 17.1 months, *p* = 0.019) [3]. It appears that Black mCRPC patients may derive a greater margin of benefit from hormone-based therapy, compared with White mCRPC patients [3].

**Figure 1:**
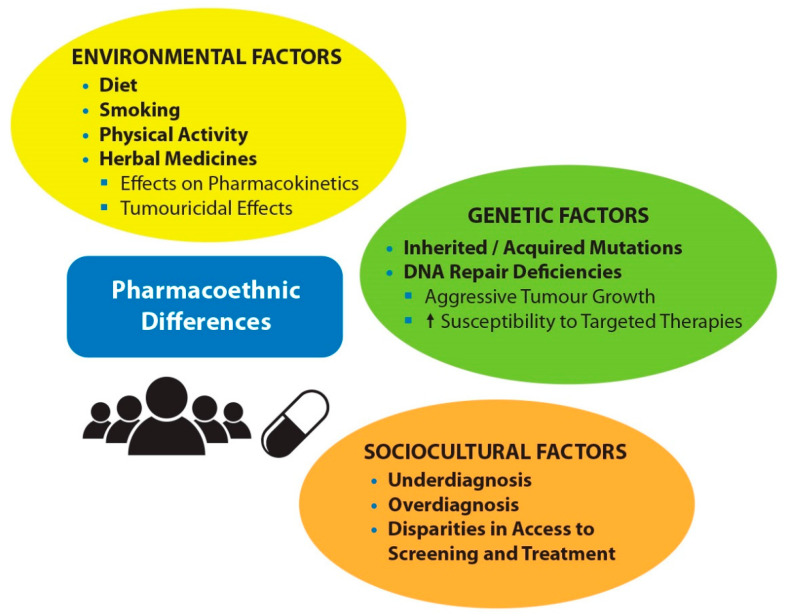
Factors that may contribute toward pharmacoethnic differences in the management of Asian mPC patients.

Ethnic diversity in the response and toxicity to drug treatment among patients with PC is thought to be multifactorial and complex, encompassing environmental, sociocultural, and genetic differences [4]. Environmental influences include diet, smoking, physical activity and the use of herbal medicines (Figure 1). Soy foods, which are popular in the Asian diet, have been associated with a 25−30% reduced risk of PC [5]. In terms of sociocultural factors, local healthcare practices may lead to underdiagnosis or overdiagnosis, as well as disparities in access to screening and treatment, for some groups of patients [1]. Perhaps not surprisingly, 5-year survival rates for localized PC increase in Asian countries with medium, high, and very high human development index, at 30.1%, 43.4%, and 70.8%, respectively [6]. With the advent of next-generation sequencing (NGS) for genomic profiling, research and data on PC genetics have exploded, which include common mutations of genes involved in the inherited form, as well as those that are triggered by environmental factors in the acquired form [7]. This review focuses on the impacts of key pharmacoethnic factors in the current management of mPC in men of Asian descent.

## 2. Contribution of Herbal Medicines to Pharmacoethnic Differences in Cancer Treatment

One environmental factor that influences pharmacology in Asian populations generally is the use of Traditional Chinese Medicine, and especially herbal medicines, many of which have been commonly used for thousands of years. In cancer patients specifically, concomitant use of herbal medicines has been shown to influence the pharmacokinetics (PKs) or potentiate the response to chemotherapeutic agents, and in some cases may counteract their toxicity. A typical example is PHY906, a traditional hot water extract of four commonly used herbs (*Scutelleria baicalensis Georgi*, *Paeonia lactiflora Pall*., *Glycyrrhiza uralensis Fisch.* and *Ziziphus jujuba Mill*., in a ratio of 3:2:2:2, respectively), which was shown to decrease the gastrointestinal toxicity of irinotecan-based chemotherapy in a phase I study [8]. In 17 advanced colorectal cancer patients treated with irinotecan, 5-fluorouracil and leucovorin, who were randomised to receive PHY906 or placebo, the incidence of diarrhoea and use of anti-diarrhoeal drugs were reduced [8]. Furthermore, in an in vitro study in mice, PHY906 also enhanced the tumoricidal effect of irinotecan [9]. The widespread use of such herbal medicines, together with other dietary or supplement intake of herbal ingredients, may contribute to the pharmacological differences observed between Asian cancer patients and other ethnic groups.

## 3. Genetic Heterogeneity in mPC

Genetics appear to play an important role in the pharmacoethnicity of treatment outcomes in advanced PC. According to the multi-institutional Dream Team study of 150 tumours from mCRPC patients, approximately 90% of patients have genomic aberrations, with 23% of mutations in genes involved in the DNA repair pathways [10,11]. Such genes have been associated with aggressive tumour growth and increased mortality from hereditary PC [12,13]. For example, *BRCA* mutations were associated with an approximately 3-fold risk of PC compared with wildtype (*p* = 0.002), and a Gleason score ≥7, reflective of poor tumour cell differentiation (85% versus 57%, *p* = 0.0002) [12]. A number of other germline deletions, including some loss-of-function mutations, have also been associated with nodal involvement, metastases or T4 stage in PC [13].

Nonetheless, these mutations also provide therapeutic opportunities. The phase III PROfound study and ongoing prospective studies of poly-ADP ribose polymerase (PARP) inhibitors in advanced PC are showing anti-tumour activity and improvements in progression-free survival (PFS), specifically in patients harbouring DNA repair pathway mutations [14]. For breast cancer, subgroup analysis has shown consistent effects of the PARP inhibitor olaparib in Asian patients, compared to the overall study population [15].

In a multi-centre U.S. study, Pritchard et al. [16] found a significantly higher incidence of germline mutations across 20 genes that mediate DNA repair: 11.8% among men with metastatic disease compared with 4.6% in men with localised disease. Most of the mCRPC germline mutations were in *BRCA2* (5.3%), *ATM* (1.6%)*, CHEK2* (1.9%), *BRCA1* (0.9%), *RAD51D* (0.4%) and *PALB2* (0.4%). Similarly, a study in Shanghai conducted germline testing of over 300 Chinese patients with different PC staging [17]. In this study, 9.8% of patients had alterations in one or more of 18 PC-related DNA repair genes, of which *BRCA2* was the most commonly mutated (6.3%). In a sequencing study of 8 PC-associated genes (*ATM*, *BRCA1*, *BRCA2*, *BRIP1*, *CHEK2*, *HOXB13*, *NBN* and *PALB2*) in 7636 unselected Japanese PC patients and 12,366 non-cancer male controls, the overall mutation prevalence was 2.9% in patients versus 0.8% in controls (including *BRCA*, 1.1% vs. 0.2%, respectively) [18].

The advent of NGS for comprehensive genomic profiling has revealed a constellation of polymorphisms involved in susceptibility and treatment response to a wide range of diseases. In a study of tumour data from 2393 patients treated at Memorial Sloan Kettering Cancer Center (MSKCC) or the Dana Farber Cancer Institute, the mutational profiles of 474 genes were examined by NGS according to race and tumour stage [7]. There were not many differences in genomic profiling between White, Black and Asian men with primary tumours. However, among those with metastatic tumours, mutations in the key tumour suppressor gene *TP53* occurred significantly more often in Asian men compared with Black or White men (62% vs. 22.5% and 36.4%, respectively). Of note, Black men, who have the highest rates of PC incidence and mortality [1], were more likely to have metastatic tumour mutations in the *AR* and DNA-repair genes, as well as actionable genetic mutations, than either White or Asian patients with metastatic disease [7].

*TP53* mutations have been associated with reduced tumour dependency on androgen receptor (AR) signalling [19]. In 94 Chinese men with localised hormone-sensitive PC, there was a high prevalence of *TP53* alterations (22%) [20]. In a prospective cohort of 239 Chinese PC patients [21], 15.9% had *TP53* mutations. Compared with wildtype, *TP53* mutations were associated with increased rate of metastases (78.9% vs. 60.2%, *p* = 0.028) and castration-resistance (68.4% vs. 42.8%, *p* = 0.004).

## 4. Drug Tolerability in Asian Patients

### 4.1. Taxane Toxicity

Although the relationships between genetic ethnic disparities and clinical outcomes in PC are somewhat unclear, the differences between Asian and White patients in chemotherapy tolerance have been well established. In our reports of real-world experience using docetaxel in Chinese patients with mHSPC as well as mCRPC, we found much higher incidences of haematological complications, particularly febrile neutropenia (FN), compared with studies in Western populations [22,23,24,25] (Table 1). Furthermore, real-world data for cabazitaxel in international compassionate use and early access programmes revealed similarly elevated rates of myelosuppression among Asian patients [26,27]. Comparatively poor marrow tolerance to chemotherapy was seen in Chinese patients from Hong Kong, as well as other Asian patients with mHSPC and mCRPC.

Higher susceptibility to docetaxel-associated myelosuppression in Asian patients is not confined to PC. Yano et al. [28] performed an integrated meta-analysis of 120 phase II and III trials of docetaxel in different cancer patients. They found that studies that were conducted within Asia were significantly associated with a higher incidence (>70%) of grades 3 and 4 neutropenia, highlighting that Asian populations, in general, are inherently more prone to myelosuppression with docetaxel, irrespective of the cancer type. The authors discussed pharmacogenomic differences in drug-metabolising enzymes and/or drug transporters as a potential explanation; however, they also pointed out that population PK studies of docetaxel demonstrated similarities in systematic clearance between Western and Japanese participants, in terms of hepatic function, serum albumin and α_1_-acid glycoprotein levels, and age. Thus, PKs may not be a major explanatory factor for such differences.

### 4.2. PKs and Clearance of Taxanes

The PKs of docetaxel have been extensively evaluated since the early 1990s. In a PK study involving nearly 600 cancer patients from 24 phase II studies, docetaxel clearance was found to be a major significant predictor for FN [29]. Could inter-ethnic differences in the PK profile of docetaxel explain the higher incidence of myelosuppression in Asian populations? Prospective PK analyses demonstrated that the estimated clearance of docetaxel was highly comparable in White and Japanese populations, suggesting that there are no obvious inter-ethnic differences in the PKs of docetaxel [30]. However, these analyses also found that docetaxel clearance is related to body surface area, among other factors, including age, hepatic function, and α_1_-acid glycoprotein levels. Based on U.S. Centers for Disease Control statistics from 2002 [31], there is an obvious, remarkable body weight difference between Whites and Asians. Among average, middle-aged adult males in the USA and China, for example, there was a difference of about 20 kg in mean body weight. Although wide variations in body weight also exist between different cities in China, generally Asian people have a comparatively smaller body build; *as a result, the marrow reserve is more limited.* This is one possible explanation for the higher risk of taxane-related haematological complications.

### 4.3. AR-Signalling Pathway Inhibitors

While Asian patients fare comparatively poorly with taxanes, their tolerance to AR agents appears to be similar to the global population. We published two reports of real-life experience using abiraterone and enzalutamide in the Hong Kong Chinese population. Apart from slightly higher rates of hypertension and peripheral oedema with abiraterone [32], and hypertension and fatigue with enzalutamide [33], our mCRPC patients experienced similar rates of severe adverse events as those in pivotal studies [34,35,36,37]. The differences might, to some extent, be attributable to our inclusion of patients with higher baseline prostate-specific antigen (PSA) levels and Gleason scores, as well as reporting differences (fatigue was physician-reported in real-life, but self-reported in trials). In general, the AR agents are tolerable in Chinese patients, with few requiring treatment discontinuation due to toxicities.

Sequencing studies have associated numerous germline variants with response to abiraterone in advanced PC patients, in genes including *CYP17A1*, *AR-V7*, *HSD3B1*, *SLCO2B1*, *SULT1E1*, and *SRD5A2*, which are involved in homologous recombination, the Wnt signalling pathway, and abiraterone metabolism [38]. Most of the studies were conducted in Caucasian populations, and further Asian data will provide further details on any pharmacogenomic differences.

In one of the studies conducted in Japanese mHSPC patients, among those who received androgen deprivation therapy (ADT; *n* = 104), *HSD3B1* heterozygous and homozygous variants were associated with a higher risk of progression versus homozygous wildtype (hazard ratio [HR] = 2.34, *p* = 0.03), but not for all-cause mortality (*p* = 0.50) [39]. Interestingly, in patients who received abiraterone (*n* = 99), *HSD3B1* heterozygous variants were associated with a lower risk of treatment failure (HR = 0.32, *p* = 0.002) and all-cause mortality (HR = 0.40, *p* = 0.04). *HSD3B1* encodes the 3β-hydroxysteroid dehydrogenase-1, which is involved in dihydrotestosterone synthesis and may be implicated in ADT resistance. These results suggest that *HSD3B1* mutations may help to predict response to AR inhibitors and ADT, and further studies are warranted.

## 5. Similar Inter-Ethnic Efficacies of Common Treatments

### 5.1. Treatments in mCRPC

The differences in tolerability of cytotoxic agents between Asian and Caucasian populations have prompted investigators to look for inter-ethnic differences in the treatment response to PC drugs. Based on data from the registration trials for docetaxel and abiraterone in China [40,41], Asian patients appear to derive a similar magnitude of survival benefit with these life-prolonging therapies when compared with the rest of the world. In 228 Chinese mCRPC patients randomised to receive 10 cycles of 75 mg/m^2^ docetaxel or mitoxantrone 12 mg/m^2^ every 3 weeks (both with oral prednisone 10 mg daily), the primary endpoint of median OS was 21.9 months in the docetaxel group versus 13.7 months in the mitoxantrone group (HR = 0.63, *p* = 0.0011) [40]. In 214 Chinese mCRPC patients randomised 2:1 to receive abiraterone acetate 1000 mg once daily or placebo (both with prednisone 5 mg twice daily), the median time to PSA progression (primary endpoint) was 5.6 months in the abiraterone arm versus 2.8 months in the placebo arm (HR = 0.51, *p* = 0.0001) [41]. In the subgroup analysis focused on East Asian populations within the PREVAIL study (61 Japanese, 78 Korean and 9 Singaporean patients), the HRs for radiographic PFS and OS (co-primary endpoints) in the enzalutamide 160 mg/day versus placebo arms were 0.38 (95% confidence interval [CI]: 0.10–1.44) and 0.59 (95% CI: 0.29–1.23) in East Asian patients, compared to 0.19 (95% CI: 0.15–0.23) and 0.71 (95% CI: 0.60–0.84) in the overall population [42].

### 5.2. Treatments in mHSPC

Despite the higher risk of myelosuppression with taxanes, the efficacies of chemotherapies in Asian mHSPC patients are comparable to those in reports from Western countries. In our multi-centre study of Chinese men in Hong Kong [43], in which >90% of patients had high-volume disease, the benefit of delaying time-to-castration-resistance with the combination of ADT + chemotherapy over that of ADT alone was similar to that found in the pivotal CHAARTED study [23]. In both studies, patients received 75 mg/m^2^ docetaxel every 3 weeks (except one elderly patient in the Hong Kong trial who received 60 mg/m^2^ docetaxel) without prednisone; approximately 94% and 86% of patients in the docetaxel arms completed 6 cycles, respectively. Chemohormonal therapy demonstrated significant associations with longer times to both PSA progression and castration resistance. However, the CHAARTED trial observed a significant difference in clinical progression (HR = 0.61 for docetaxel vs. placebo, *p* < 0.001), whereas our study did not, possibly due to our relatively short follow-up period in the chemohormonal group. These findings further suggest that pharmacoethnic differences in Asian patients are largely limited to the toxicity of the drug class, rather than the response to it.

## 6. Risk Management of Taxane-Related Myelosuppression in Asian PC Patients

**Figure 2:**
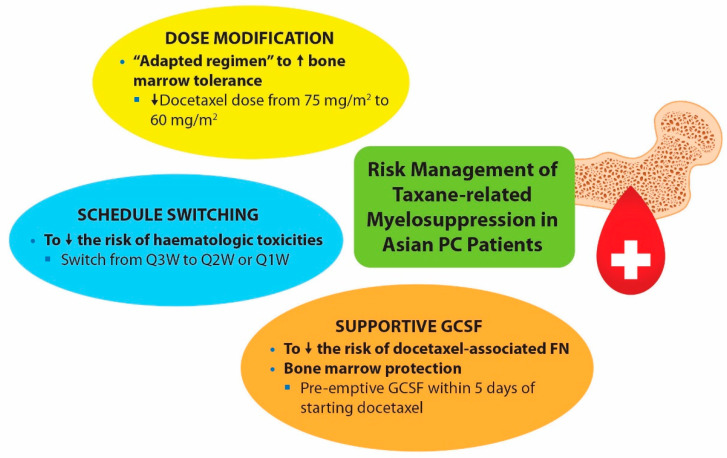
Strategies for reducing the risk of taxane-related myelosuppression in Asian prostate cancer (PC) patients. GCSF, granulocyte colony-stimulating factor; FN, febrile neutropenia.

While we await further data to explain the mechanisms underlying inter-ethnic differences in pharmacology, there are several approaches to address the higher risk of taxane-related myelosuppression in our patients (Figure 2). These include modification of the dose (75 mg/m^2^ to 60 mg/m^2^) or scheduling frequency (Q3W to Q2W/Q1W), as well as pre-emptive use of granulocyte colony-stimulating factor (GCSF).

### 6.1. Dose Modification

In view of the higher risk of myelosuppression, dose modification or reduction of docetaxel is already a common practice for the management of mCRPC patients in Asia. In a retrospective study in Japan, a reduced-dose or so-called “adapted regimen” of lower-dose docetaxel was compared with a standard regimen of 60 mg/m^2^ every 4 weeks in patients with CRPC and well-balanced baseline characteristics [44]. The adapted regimens consisted of three different administration patterns: (1) 48 mg/m^2^ Q4W from the start; (2) starting with 60 mg/m^2^ Q4W, followed by 48 mg/m^2^ Q4W when not tolerated; or (3) starting with 30 mg/m^2^ Q2W for a few weeks, followed by 48 mg/m^2^ Q4W. Patients on the adapted regimen (*n* = 29) demonstrated similar toxicities, PSA response and survival outcomes compared with those on the standard regimen (*n* = 28).

Another retrospective analysis from Singapore compared outcomes of CRPC patients who received docetaxel at 75 mg/m^2^ Q3W (*n* = 11) with those who received 60 mg/m^2^ Q3W (*n* = 38) and 20 or 35 mg/m^2^ weekly (*n* = 40) [45]. Patients who received 75 mg/m^2^ Q3W were younger than those who received 60 mg/m^2^ Q3W, and patients who received weekly regimens were more symptomatic (*p* = 0.02) than those who received 60 mg/m^2^ Q3W. Accordingly, 73%, 50% and 46% of patients in the three groups showed >50% fall in PSA from baseline, but the differences were non-significant. However, OS was significantly longer in the 60 mg/m^2^ Q3W than the 20 or 35 mg/m^2^ weekly group (16.9 vs. 10.6 months, *p* = 0.01).

The above results suggest that a reduced-dose regimen may be feasible, offering a similar clinical outcome as the standard regimen in Asian PC patients. The Japan study authors also noted that an objective quality-of-life assessment would be helpful for evaluating patients’ well-being.

At the Advanced Prostate Cancer Consensus Conference (APCCC) 2019, 72 PC experts met to discuss areas of controversy in patient management, including four representatives from Asia: one each from Hong Kong, India, Japan and Singapore [46]. One question considered the starting dose to initiate treatment with a taxane for mCRPC patients of East Asian descent. Given the concern regarding poor marrow tolerance to chemotherapy in Asian PC patients generally, more than half (60%) of the respondents preferred to start with a reduced dose of taxane, with possible consideration of dose escalation or further reduction, as indicated.

### 6.2. Schedule Switching

Switching the schedule from every 3 weeks to every 2 weeks is another potential approach to reducing chemotherapy-related haematological toxicities. In the randomised phase III study published in *The Lancet Oncology* in 2013 [47], 50 mg/m^2^ docetaxel administered every 2 weeks was associated with better time to treatment failure (the primary endpoint) compared with the standard 3-weekly schedule, at 5.6 versus 4.9 months (HR = 1.3, *p* = 0.014). OS was also slightly longer, at 19.5 versus 17.0 months (HR = 1.4, *p* = 0.021). While commentators noted that the study was not powered to assess this survival outcome, and only approximately one-fifth of patients in each group received second-line chemotherapy [48], it is important to note that the 2-weekly schedule was associated with fewer haematological toxicities. Only 4% of the 2-weekly patients developed FN, compared with 14% of the 3-weekly patients, and grade 3–4 neutropenia and leukopenia were also significantly less frequent in the 2-weekly arm.

A recently published phase II trial of 2-weekly docetaxel in 42 Korean mHSPC patients reported a median CRPC-free survival of 26.4 months, with a 1-year CRPC-free rate of 79% [49]. Anaemia occurred in 95% of patients, followed by nail change (33%), fatigue (29%) and oral mucositis (26%). No FN developed during the 25-month median follow-up. Although comparison data from a randomised trial are not yet available for Asian patients, the 2-weekly schedule could be one way to reduce the incidence of docetaxel-related myelosuppression.

### 6.3. Supportive GCSF

On behalf of the Hong Kong Society of Uro-Oncology (HKSUO), our team reviewed the incidence of docetaxel-related haematological toxicities in mPC patients treated in six public oncology centres in Hong Kong [50]. Among 377 Chinese patients who received docetaxel, the overall incidence of FN was 15%; those with mCRPC had a higher incidence (17%) compared with that in the mHSPC patients (12%). In this cohort, 29% of the patients received a reduced starting dose of docetaxel, and 18% received pre-emptive GCSF within 5 days (2 and 69 patients with and without 1st FN, respectively). The use of pre-emptive GCSF significantly reduced the incidence of docetaxel-associated FN overall, with similar trends in both groups.

Bone marrow protection with pre-emptive GCSF was also seen in a Japanese population of patients treated with cabazitaxel. In a prospective study that evaluated pre-emptive GCSF (pegfilgrastim) in every cycle of cabazitaxel, only 9% of the 21 mCRPC patients developed FN [51]. This result was in stark contrast to the 54% of patients who developed FN without GCSF intervention in a phase I study of cabazitaxel in Japanese mCRPC patients (*n* = 44) [52].

Based on these studies, it is now common clinical practice in Hong Kong to consider the use of pre-emptive GCSF when docetaxel is administered to patients with metastatic disease. We also suggest careful patient selection for docetaxel, which should include considerations of disease volume, performance status (rather than age), and exclusion criteria such as severe hepatic impairment, grade ≥2 neuropathy, and platelets <50 × 10^9^/L and/or neutrophils <1.0 × 10^9^/L (as in the APCCC 2017 consensus [53]). Both of these practices are recommended in a 2018 joint consensus statement issued by the Hong Kong Urological Association (HKUA) and the HKSUO [54].

## 7. COVID-19 Considerations

How may the above-discussed pharmacoethnic considerations be applied in the midst of the COVID-19 outbreak? On the one hand, mPC patients may require extra care because of the elevated risk of infection from an immune-suppressive state, which may be the result of treatment-related AEs, reduced physical performance status, and old age [55]. Studies suggested that among COVID-positive cancer patients, those with PC were more likely to be hospitalized or die than those with non-prostate genitourinary malignancies [56]. On the other hand, physical contact may need to be minimised to reduce the risk of transmission, such as in terms of postponing surgeries and limiting in-person consultations [57].

A joint consensus of the HKSUO and HKUA was recently formulated to address the management of ethnic Chinese PC patients during COVID-19 [55]. The consensus noted that chemotherapy can result in immunosuppression that increases the risks of fever and infection. Although most would agree that oral AR agents are preferable in the COVID-19 setting, chemotherapy remains an essential treatment option in metastatic disease in Hong Kong and other Asian countries, where it is comparatively less expensive. Given the higher risk of taxane-related haematological complications in our patients, we recommend the pre-emptive use of GCSF with taxanes to reduce the risk of neutropenia, which makes patients more susceptible to viral infections. However, note that schedule-switching can increase hospital visits [58], and should be applied on a limited basis.

## 8. Discussion and Future Directions

There are clear inter-ethnic differences in the pharmacology, and especially cytotoxicity, of agents used in the treatment of PC. These differences may result from the living environment (e.g., diet and exercise), everyday practices (e.g., use of herbal medicines), access to health care (e.g., treatment selection), and genetic factors.

Arguably, genetic variations have direct effects on treatment outcomes, because they act at the molecular level and can be profiled. The study of these variations will require large-scale sequencing and data collection efforts. Current data have yet to reveal any distinct disparities in genomic profiling between Asian and White PC patients. Zhu et al. [59] noted that precision medicine in PC consists of targeting the following major pathways: AR signalling, PTEN-PI3K-AKT signalling, DNA damage repair, cell cycle regulation, and lineage plasticity (e.g., loss of AR signalling and activation of neuronal and neuroendocrine pathways) [60]. Given the observed differing treatment responses between Asian and Western populations, treatment optimisation can be exercised on genetic alterations that are present along these pathways. Much more work on the characterisation of these variants is warranted and may provide a deeper understanding of resistance mechanisms.

Going forward, we must ask how the complex interplay between environmental, lifestyle and genetic factors might be reflected in the tolerance and response to treatment? How can we further tailor our management strategy to meet the needs of Asian patients? How can future clinical trials and precision oncology studies be designed to adequately represent all ethnic groups?

Given the observed differences, there appears to be an under-representation of Asian patients in clinical trials of PC treatments. Subsequent local studies (e.g., our above-mentioned Hong Kong studies) using different patient inclusion/exclusion criteria and assessment methodologies may produce somewhat different results from the original registration trials. *Post hoc* subgroup analyses have been helpful for confirming the effects on Asian populations, as in PREVAIL [42], but they are often not sufficiently powered to detect specific differences. PK studies could clarify whether any pharmacoethnic differences are the result of drug clearance or other variations along the targeted biochemical pathways.

While the past decade has seen the development of a variety of new treatment options for mPC patients, treatment sequencing has become increasingly important and is highly contingent on response and tolerability. A detailed understanding of pharmacoethnic differences can facilitate fine-tuning of treatment sequencing and dosages. Because PC patients are generally older, a treatment sequencing strategy aimed at preserving their physical performance may minimise the risk of AEs including FN. Moreover, the preliminary observation that patients with certain genetic variants (e.g., *HSD3B1*) may be more responsive to certain therapies than others may affect treatment sequencing recommendations.

As mentioned, there is increasing evidence that germline mutations contribute to the risk of PC, some of which are also actionable [61]. Repositories of clinical and genomic data, such as the Prostate Cancer Precision Medicine Multi-Institutional Collaborative Effort (PROMISE) [62], will help to enable precision oncology. Local and regional data will be needed to document relevant hotspot mutations, and to characterise variants of uncertain significance, perhaps in a manner similar to those for hereditary breast and ovarian cancers [63].

## 9. Conclusions

The elucidation of ethnic and pharmacogenomic differences in PC may lead to a better understanding of the aetiology and risk factors influencing prognosis and response to therapy for this all-too-common disease. While the treatment outcomes for taxanes such as docetaxel are similar worldwide, there are higher incidences of haematological complications, particularly myelosuppression, in Asian populations. There are no obvious inter-ethnic differences in the PKs of docetaxel that might explain this phenomenon. While we await clarification on the mechanisms underlying pharmacoethnic differences, several approaches can be considered to mitigate the higher risks of taxane-related myelosuppression immediately: namely, modification of dosing and/or scheduling, and pre-emptive use of GCSF. The comparatively smaller body build and limited marrow reserve in Asian men may underlie the need for adjusted dosing of taxanes. By contrast, AR agents are generally tolerable in Asian PC patients.

## Figures and Tables

**Table 1:** Comparison of grade 3 and 4 adverse events (AEs) related to taxane treatment in studies of Asian and Western populations.

Population	mHSPC	mHSPC	mCRPC	mCRPC	mCRPC	mCRPC
Study Location	Hong Kong [22]	USA (CHAARTED Study) [23]	Hong Kong [24]	UK (TAX-327 Study) [25]	Asia-Pacific * (CUP/EAP Study) [26]	Europe (CUP/EAP Study) [26]
Grade 3/4 Adverse Events (%)						
Febrile neutropenia	12.5	6.2	14.1	3.0	15.1	4.8
Neutropenia	40.6	12.1	47.4	32.0	27.3	17.1
Thrombocytopenia	0	0.3	0	1.0	1.7	1.0
Anaemia	3.1	1.3	10.6	5.0	12.2	3.1
Neuropathy	0	0.5	0	0	N/A	N/A
Fatigue	0	4.1	0	5.0	4.7	6.8
Diarrhoea	0	1.0	1.8	0	6.4	3.0
Stomatitis	0	0.5	1.8	0	N/A	N/A

CUP/EUP = pooled analysis of cabazitaxel compassionate use and early access programmes; mHSPC/mCRPC = metastatic hormone-sensitive/castration-resistant prostate cancer, respectively; N/A = not available. * Includes Australia, Bangladesh, Taiwan, India, Kazakhstan, South Korea, Malaysia, Philippines, Singapore and Thailand.
